# Electropolishing influence on biocompatibility of additively manufactured Ti-Nb-Ta-Zr: in vivo and in vitro

**DOI:** 10.1007/s10856-023-06728-0

**Published:** 2023-05-14

**Authors:** J. P. Luo, K. P. Lv, J. C. Tang, Z. Z. Wu, Y. L. Liu, J. T. Luo, Y. X. Lai, M. Yan

**Affiliations:** 1 Centre for Translational Medicine Research & Development, Shenzhen Institutes of Advanced Technology, Chinese Academy of Sciences, Shenzhen, 518055 China; 2 Department of Materials Science and Engineering, Southern University of Science and Technology, Shenzhen, 518055 China; 3 Shenzhen Distinta Interfacial Technology Co. Ltd, Shenzhen, 518106 China; 4 Department of Interventional Radiology, Shenzhen People’s Hospital (The Second Clinical Medical College, Jinan University; The First Affiliated Hospital, Southern University of Science and Technology), Shenzhen, 518020 China; 5 School of Physics and Opto-electronic Engineering, Shenzhen university, Shenzhen, 518060 China; 6 Jiaxing Research Institute, Southern University of Science and Technology, Jiaxing, 314001 China

## Abstract

Balling defect of the additively manufactured titanium lattice implants easily leads to muscle tissue rejection, which might cause failure of implantation. Electropolishing is widely used in surface polishing of complex components and has potential to deal with the balling defect. However, a clad layer could be formed on the surface of titanium alloy after electropolishing, which may affect the biocompatibility of the metal implants. To manufacture lattice structured β-type Ti-Ni-Ta-Zr (TNTZ) for bio-medical applications, it is necessary to investigate the impact of electropolishing on material biocompatibility. In this study, animal experiments were conducted to investigate the in vivo biocompatibility of the as-printed TNTZ alloy with or without electropolishing; and proteomics technology was used to elaborate the results. The following conclusions were drawn: (a) a 30% oxalic acid electropolishing treatment was effective in solving balling defects, and ~21 nm amorphous clad layer would be formed on the surface of the material after polishing; (b) the electropolished TNTZ suggested decreased cell cytotoxicity and improved blood biocompatibility as compared to as-printed TNTZ; (c) the amorphous clad layer could make a barrier to prevent Ta and Zr ions from penetrating into the muscle tissue, and could form a good tissue regeneration at the implantation site during 4 weeks, indicating that the electropolished TNTZ has the potential as implants; and (d) the cells attached to the electropolished TNTZ showed higher antioxidant capacity but less proliferation than attached to as-printed TNTZ.

**Graphical Abstract:**

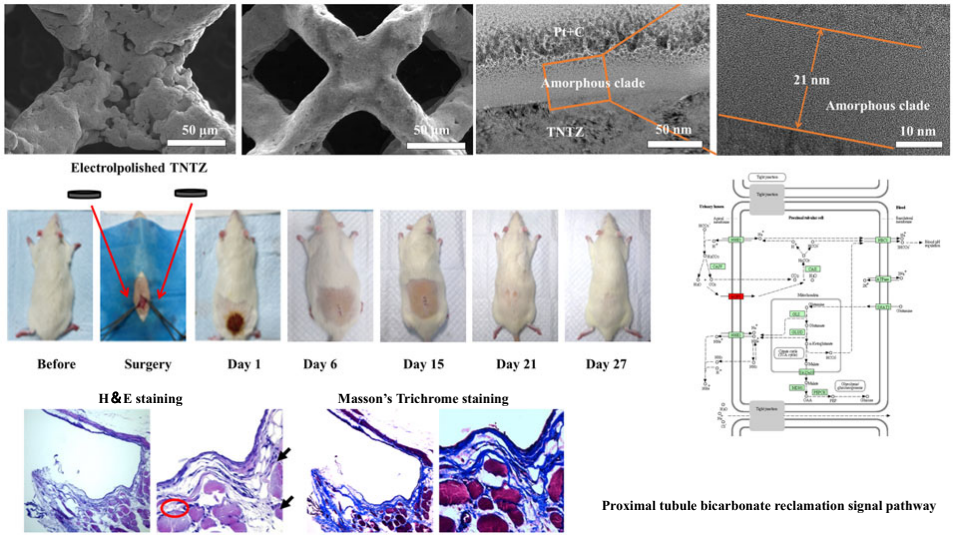

## Introduction

Low-modulus β titanium alloys have been widely studied for their superior biocompatibility, higher preparation efficiency and easier customization, which make them suitable for making as-printed lattice implants [1, 2]. However, some studies have rececntly shown that the balling defects of the additively manufactured (as-printed) lattice implants lead to pathological issues in adjacent tissues, eventually, resulting in failure of implants [3]. To decrease the influence of balling defects, electropolishing was tried to treat as-printed lattice implants [4]. Some studies have shown that electropolishing changed the surface chemical properties of the as-printed metal implants, which may improve the biocompatibility of β titanium alloy implants [5]. However, related researches mainly focused on corrosion resistance and in vitro cytotoxicity tests. Mechanism relating to in vivo inflammatory responses was rarely reported [6].

As a typical β titanium alloy, Ti-30.4Nb-5.28Ta-7.16Zr (nominal composition of Ti-30Nb-5Ta-8Zr, wt%) has been proved to have low Young’s modulus, nice printability and good biocompatibility in previous studies [7]. In this work, animal experiments were conducted to investigate the in vivo biocompatibility and systemic inflammatory responses of the as-printed TNTZ alloy lattice with or without electropolishing. Besides, proteomics technology was performed to analyse the molecular mechanisms of the results.

## Experimental

### Raw material preparation

An EOS M290 3D printer equipped with a 400 W Yb: YAG continuous wavelength fiber laser (~70 μm beam size) was used to fabricate the samples under a high-purity Ar atmosphere. The TNTZ powders were manufactured by Plasma Rotating Electrode Process (PREP) (supported by Xian Sailong Company, a spin-off of the Northwest Institute for Nonferrous Metal Research). The TNTZ discs were additively manufactured through the processing parameters of laser power (*P*, 240 W), layer thickness (*D*, 50 μm), and scanning speed (*v*, 900 mm/s) [8]. The stl. file of the lattice sample was generated by the MAGICS software (Materialise, Leuven, Belgium), then the lattice sample was also additively manufactured by SLM. All the samples mentioned in this work were annealed (at 600 °C, 3 h) for stress relief, and were listed in Table 1. To facilitate electropolishing, cell culture and animal experiments, all discs were preliminarily mechanical polished to a similar roughness.

**Table 1:** List of the various types of as-printed samples and the number of times their properties were repeatedly tested

Category	Specifications	Number of discs	
		Polished group	Control group
Electropolishing	Ø13 mm × 0.8 mm	36	0
Implant	Ø2 mm × 0.8 mm	6	6
Biocompatibility	Ø13 mm × 0.8 mm	20	20
Proteomics	Ø75 mm × 0.8 mm	3	3
Total		23	23

### Electropolishing

According to the literature [9], oxalic acid could form soluble metal salts with metal ions as a protective layer to prevent corrosion on the anode surface; and as a solvent, acetic acid could not be directly involved in the reaction. In this work, an electropolishing device was independently developed and an electrolyte prepared with 30% oxalic acid +70% acetic acid was used for electropolishing.

To quantitatively characterize the effect of different process parameters on electropolishing, electropolished discs (3 discs in 1 group) were used to study the process from four aspects such as current density, polishing spacing, temperature and immersion time. The three levels of process parameters were shown in Table 2. Based on the electropolishing results, half of the remaining discs were selected for electropolishing.

**Table 2:** Factor levels table of orthogonal experiments

Factor	Current density (A/cm^2^)	Polishing spacing (mm)	Temperature (°C)	Immersion time (min)
Level 1	0.7	10	15	90
Level 2	1.05	15	20	120
Level 3	1.4	20	30	150

### Surface characterization and hardness measurement

Surface roughness of the samples was measured by a 3D profiler (Bruker, ContourTG-K0, Germany). Five measurements with a scan area of 0.09 × 0.12 mm^2^ were performed at random positions on 3 samples.

The thickness of the clad layer was thinned by Focused Ion Beam (FIB, FEI Helios 600i, America), with the parameter 30 kV, 0.79 nA (1000 nm), 0.23 nA (300 nm), 80pA (120 nm), and calibrated by Transmission Electron Microscope (TEM, Hitachi HT7700, Japan) Pt and C was selected as protection layer and calibrated by Transmission Electron Microscope (TEM, Hitachi HT7700, Japan). The surface composition was analyzed using X-ray Photoelectron Spectroscopy (XPS, Thermo Scientific Escalab 250X, America), with an Al-K_α_ X-ray source (1486.6 eV) at a take-off angle of 90°.

The Vickers hardness was measured on the polished samples through a microscopic Webster hardness Tester with a 500 N load and a dwell time of 15 s (diamond indenter φ 2.8 mm). An average of 12 points was tested for each sample; the deviation in these measurements was less than ±0.3 %.

### In vitro cell compatibility test by CCK-8 Kit

All discs were cleaned using acetone for 15 min in an ultrasonic bath followed by rinsing in 75% alcohol (20 min, twice) and subsequently in ultrapure water (20 min, twice). After ultrapure washing, the discs were sterilized at 200 °C and then dried at 60 °C for 24 h. The preparation of the extract was made by the relevant provisions of ISO 10993-1:18 on cytotoxicity testing [10]. After high temperature and high-pressure disinfection, the as-printed and electropolished TNTZ were immersed in serum-containing culture medium at the ratio of 0.2 g sample/mL, and placed in a 37 °C shaker to extract for 24 and 48 h, respectively, biosafety of the material extracts solution was tested by CCK-8.

The L929 cell line was purchased from the Cell Bank of Type Culture Collection of Chinese Academy of Sciences (Shanghai, China). L929 cells were seeded into a 96-well plate at a density of 5 × 10^4^ cells/mL (100 μL/well), and cultured in 1640 culture medium (89% 1640 medium, 10% Fetal Bovine Serum (FBS), 1% double-resistance penicillin and streptomycin) overnight in a 5% CO_2_ incubator at 37 °C condition overnight. Then the culture medium was replaced by the given different types of material extracts solution and 6 wells were contained in each group After 24 h, the culture medium was removed and washed twice with PBS. An amount of 100 μL of culture medium with 10% CCK-8 was added and cultured for 2 h. The Optical Density (OD) value was measured at 450 nm. The relative cell viability was calculated according to the following formula. Cell viability (%) = [OD value of the experimental group/OD value of the negative control group] × 100%.

### Hemolysis test

The in vitro hemolysis of the TNTZ was determined by hemoglobin released from erythrocyte when the TNTZ was in direct contact with blood, reference to Li [11]. C57BL/6 (male) of conventional grade (GEMPHARMATECH, Foshan. China) 8 weeks was used to collect blood. The blood was collected by picking out the eyeball with anticoagulant tube. Four times volume of sodium chloride injection was added into the fresh anticoagulant blood to prepare erythrocyte suspension. The suspension was centrifuged at 1500 rpm for 15 min, and supernatent was removed. The precipitated red blood cells were washed and centrifuged 2 times with sodium chloride injection to obtain red blood cells. In the experimental group (as-printed TNTZ and electropolished TNTZ), 5 discs and 5 mL sodium chloride injection (twice mass of the sample) were added into the test tube. In negative control group (NC group), 10 mL sodium chloride injection was added into the test tube. In positive control group, 10 mL distilled water was added into the test tube. All the test tubes were immersed in thermostatic water bath at 37 °C for 30 min, and then added 0.2 mL red blood cells into the test tubes. After blending, the test tubes were immersed in thermostatic water bath at 37 °C for 60 min continually.

Finally, the liquid in the test tubes was collected and centrifuged at 1500 rpm for 5 min, and supernatant fluid was removed into cuvette. The OD of the supernatant fluid was quantified photometrically using a Victor1420 (PerkinElmer Company, USA) at the wavelength of 570 nm. The test was replicated three times. Hemolytic properties were determined by hemolysis ratio. The hemolysis ratio (Z) was calculated using the following equation: Z(%) = [(ODt−ODnc) ODpc−ODnc] × 100%. ODt refers to the mean value of the measured optical density of the experimental group, ODnc refers to the mean value of the measured optical density of a negative control group, and ODpc refers to the mean value of the measured optical density of a positive control group.

### Calcein AM/PI staining

Different discs (Ø13 mm × 0.8 mm) were placed on 24-well plates, each group contained 3 repeated wells. Then L929 cells were seeded in a 24-well plate at a density of 1 × 10^4^ cells/well (500 μL/well), and cultured overnight in a 5% CO_2_ incubated at 37 °C. The time gradient for the experiment was 1, 4 and 6 days respectively, changing the medium every other day, when time over the AM/PI staining experiment was carried on. In accordance with the instructions of Calcein/PI Cell Viability/ Cytotoxicity Assay Kit (Beyotime Biotechnology, China), the cells were gently washed with PBS two or three times to ensure that the active esterase contained in the medium must be removed. 250 μL Calcein AM/PI working fluid (AM 1X and PI 1X) was added to each well. After incubation at 37 °C for 20 min, the cells were observed and photographed through confocal high-content analysis system of cell imaging.

### Animal experiments

#### Ethics statement

All animal experiments in this work were conducted in accordance with the recommendation of the Guide for the Care and Use of Laboratory Animals of the National Institutes of Health. Our implantation tests were under the approval of the Ethics Committee of Shenzhen People’s Hospital and the Animal Management Committee of the Southern University of Science and Technology. All efforts were made to minimize the animals’ suffering during the surgeries.

#### Subcutaneous implantation of metal sheets

Specific Pathogen-Free (SPF) grade mature and healthy male Sprague Dawley (SD) rats weighing from 300 to 320 g were purchased from GEMPHARMATECH (Foshan, China) and maintained at Shenzhen People’s Hospital Translational Medicine Collaborative Innovation Center (Shenzhen, China). Implantation was conducted at the bilateral gluteal muscle of the SD rats using a well-established and previously reported surgical method [12]. Before surgery, the SD rats were housed for 1 week for acclimatization. After that, all the SD rats were placed on an operating table, and 45 mg/kg 2% (w/v) pentobarbital sodium (Merck, Darmstadt, Germany) was provided as an anesthetic via intraperitoneal injection. After successful anesthesia, the hair of the implanted area on the back was shaved and the surgical area was sterilized with 10% povidone iodine. The gluteus skin was cut along the midline, and the bilateral gluteus muscles wass exposed. Separated along the direction of the muscle fibers to make a small muscle bag about 7 × 7 × 7 mm in size, and then implanted the material, the control group was the same surgical procedure but without any implant. A total of 2 discs (Ø2 mm × 0.8 mm) were separately implanted into the back of each rat. The subcutaneous tissue and skin were sutured with 4–0 Nylon, and the wound was sterilized with aqueous iodine. After the implanting surgery, postoperative SD rats were separated to be fed with a regular diet and the healing of the surgical incision was regularly observed.

#### Enzyme-linked immunosorbent assay (ELISA) analysis

After 2, 16, and 27 days of implantion, the SD rats were anesthesia tied up with hemp ropes to fix their limbs on the operating table, 1.5 mL of blood was collected from each rat through the tail vein. Blood plasma was collected by a blood collection vessel containing an anticoagulant. Then, blood samples were centrifuged at 1000 g for 10 min, and supernatant was divided into EP tubes and frozen at –20 in refrigerator for ELISA detection. In this study, a total of 4 indexes of TNF-α, IL-10, IL-6 and IL-1β were detected.

#### Histological analysis following implantation of metal sheets

After 4 weeks of implantation, the experimental animals were euthanized by overdose anesthesia. Heart, liver, spleen, lung and kidney were harvested, and the implanted samples with surrounding soft tissue were cut together. For the control group, the surgical point was taken as the center, and the surrounding tissue was taken. All the samples were fixed in 4% paraformaldehyde for about one day, then embedded in paraffin. The processed specimens’ sections were stained with Hematoxylin/ Eosin (HE) and Masson’s trichrome staining. The histological images of fibrous capsulation around implants were observed and captured with a fully automatic inverted fluorescence microscope (Dmi8 + DFC7000T, leica, Germany).

### Ion release analysis

In total, 0.5 g of leg tissue was cut from each rat, and digested by 90 °C water bath heating for 1.5 h (the solution was 30 mL 2% HNO_3_). The release of Ti, Nb, Ta and Zr ions from each experimental group was measured by Inductively Coupled Plasma Mass Spectrometry (ICP-MS).

### Proteomic analysis

#### Protein extraction and digestion

All proteomic discs (Ø75 mm × 0.8 mm) were cleaned in acetone followed by 75% alcohol (20 min, twice), and ultrapure water (20 min, twice). Then all discs were sterilized by high temperature (200 °C, 3 h) and dried (60 °C, 24 h). The discs were placed in TCPS dish separately. L929 cells were seeded at a density of 3.6 × 10^4^ cells/cm^2^ on these discs and cultured at 37 °C in 5% CO_2_ + 95% air atmosphere for 48 h, respectively. Cells cultured on TCPS surfaces were set as control. After the incubation, the medium of each group was removed, and the samples were gently rinsed (1 min) twice in PBS to remove the residual medium, and then samples were placed in new dishes to avoid interference from serum proteins adsorbed on TCPS. The adherent cells on each disc were scraped separately by cell scraper and transferred to 15 mL centrifuge tube before cell protein extraction.

300 mL SDT (4% SDS, 100 mM Tris-HCl, 1 mM DTT, pH 7.6) buffer was added to each tube (about 200 mg proteins in each tube) and heated in boiling water bath for 15 min to extract the proteins in the cells. The amount of protein was quantified with the Bicinchoninic Acid (BCA) Protein Assay Kit (Bio-Rad, USA). Protein digestion by trypsin was performed according to Filter-Aided Sample Preparation (FASP) procedure described by Matthias Mann. The digest peptides of each sample were desalted on C18 Cartridges (Empore™ SPE Cartridges C18 (standard density), bed I.D. 7 mm, volume 3 mL, Sigma), and after freeze-drying, 40 μL 0.1% formic acid solution was added for re-solubilization. For each protein sample, the experiments were carried out in triplicates.

#### LS-MS/MS analysis

LC-MS/MS analysis was performed on a timsTOF Pro mass spectrometer (Bruker) that was coupled to Nanoelute (Bruker Daltonics) for 60 min. The peptides were loaded onto a reverse phase trap column (Thermo Scientific Acclaim PepMap100, 100 μm×2 cm, nanoViper C18) connected to the C18-reversed-phase analytical column (Thermo Scientific Easy Column, 10 cm long, 75 μm inner diameter, 3 μm resin) in buffer A (0.1% Formic acid) and separated with a linear gradient of buffer B (84% acetonitrile and 0.1% Formic acid) at a flow rate of 300 nL/min controlled by IntelliFlow technology. The mass spectrometer was operated in positive ion mode. The mass spectrometer collected ion mobility MS spectra over a coefficient range of 100–1700 (m/z), 0.6–1.6 (1/K0), and then performed 10 cycles of PASEF MS/MS with a target intensity of 1.5k and a threshold of 2500. Active exclusion was enabled with a release time of 0.4 min.

The MS raw data for each sample were combined and searched using the MaxQuant 1.5.3.17 software for identification and quantitation analysis. Related parameters and instructions were shown in Table 3.

**Table 3:** Maxquant identification and quantitation indexes

Item	Value
Enzyme	Trypsin
Max Missed Cleavages	2
Fixed modifications	Carbamidomethyl (C)
Variable modifications	Oxidation (M)
Main search	6 ppm
First search	20 ppm
MS/MS Tolerance	20 ppm
Database	Swissprot_mouse_17097_20220104.fasta
Database pattern	Reverse
Include contaminants	True
Protein FDR	≤0.01
Peptide FDR	≤0.01
Peptides used for protein quantification	Use razor and unique peptides
Time window (match between runs)	2 min
Protein quantification	LFQ
Min. ratio count	1

#### Bioinformatic analysis

Cluster 3.0 [http://bonsai.hgc.jp/~mdehoon/software/cluster/software.htm] and Java Treeview software [http://jtreeview.sourceforge.net] was used to perform hierarchical clustering analysis. Euclidean distance algorithm for similarity measure and average linkage clustering algorithm (clustering uses the centroids of the observations) for clustering were selected when performing hierarchical clustering.

The protein sequences of the selected differentially expressed proteins were locally searched using the NCBI BLAST + Client software and InterProScan to find homolog sequences, then Gene Ontology (GO) terms were mapped and sequences were annotated using the Blast2GO software. The GO annotation results were plotted. Following annotation steps, the studied proteins were blasted against the online Kyoto Encyclopedia of Genes and Genomes (KEGG) database [http://geneontology.org] to retrieve their KEGG orthology identifications and were subsequently mapped to pathways in KEGG.

Enrichment analyses were applied based on the Fisher exact test, considering the whole quantified proteins as a background dataset. Benjamini- Hochberg correction for multiple testing was further applied to adjust derived p-values. And only functional categories and pathways with p-values under a threshold of 0.05 were considered as significant.

### Statistical analysis

All the data were reported as mean ± standard deviation. The statistical analysis between different groups was determined using one-way analysis of variance (ANOVA) tests. A statistically significant difference was accepted at *p* < 0.05 as usual.

## Results

### Electropolishing

**Fig. 1:**
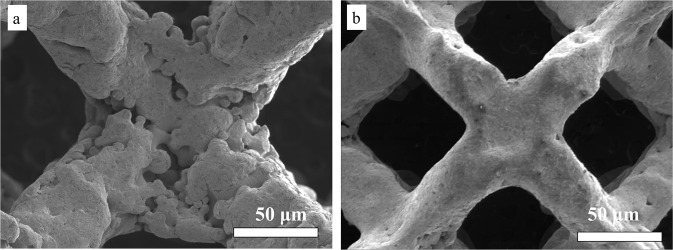
The SEM images of as-printed TNTZ lattices (**a**) before, and (**b**) after electropolishing. The corresponding parameters were immersion time 2 h, current density 1.05 A/cm^2^, polishing spacing 20 mm, and temperature 20 °C

The test results were shown in Table 4. By using the intuitive analysis method to compare the range, it was found that when the voltage remains certain, the current density has a great effect on the polishing effect, followed by the polishing spacing, temperature, and immersion time. To obtain the lowest roughness (Ra value), the optimal process parameter combination was based on the level with the lowest mean among the factors, which was finally determined as: current density 1.05 A/cm^2^, polishing spacing 20 mm, temperature 20 °C, and immersion time 2 h. Figure 1 illustrates that electropolishing with the final parameters effectively eliminate the balling defect of the as-printed TNTZ lattices. The following electropolishing experiments were carried out with the final parameters.

**Table 4:** The results of orthogonal experiment

Mode number	Current density (A/cm^2^)	Polishing spacing (mm)	Temperature (°C)	Immersion time (min)	Roughness (nm)
1	0.7	10	15	90	562
2	0.7	15	20	120	493
3	0.7	20	30	150	595
4	1.05	10	20	90	582
5	1.05	15	30	120	543
6	1.05	20	15	150	485
7	1.4	10	15	120	481
8	1.4	15	20	150	400
9	1.4	20	30	90	493
10	0.7	10	30	150	578
11	0.7	15	15	90	590
12	0.7	20	20	120	463
13	1.05	10	30	120	408
14	1.05	15	15	150	335
15	1.05	20	20	90	376
16	1.4	10	20	150	497
17	1.4	15	30	90	511
18	1.4	20	15	120	430
Mean value 1	547	518	481	519	
Mean value 2	455	479	468	470	
Mean value 3	469	474	521	482	
Range	92	44	53	49

### Surface analysis

#### Surface roughness and hardness

**Fig. 2:**
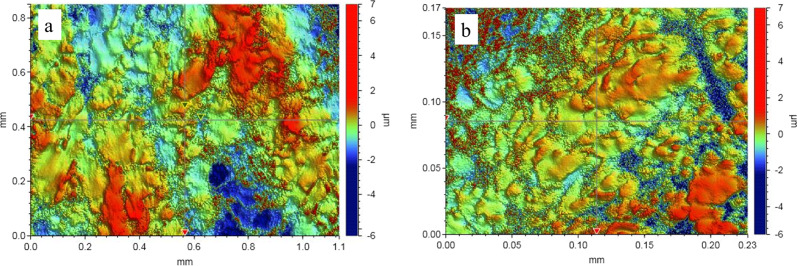
The 3D profiler images of (**a**) as-printed TNTZ, (**b**) electropolished TNTZ

The 3D profiler images (Fig. 2a, b) show that the roughness of as-printed TNTZ could be effectively reduced by electropolishing. The original sharp grooves were electropolished to a flat basin surface. The average roughness of the electropolished TNTZ (Ra ~485 nm) was much lower than that of as-printed TNTZ (Ra ~596 nm).

To reduce the influence of surface roughness, the as-printed TNTZ were mechanically polished again until Ra ~490 nm, which was equivalent to the roughness of electropolished TNTZ. Hardness tests showed that the hardness of the electropolished TNTZ was ~418HV, which was slightly harder than as-printed TNTZ (~315HV).

#### Surface composition

**Fig. 3:**
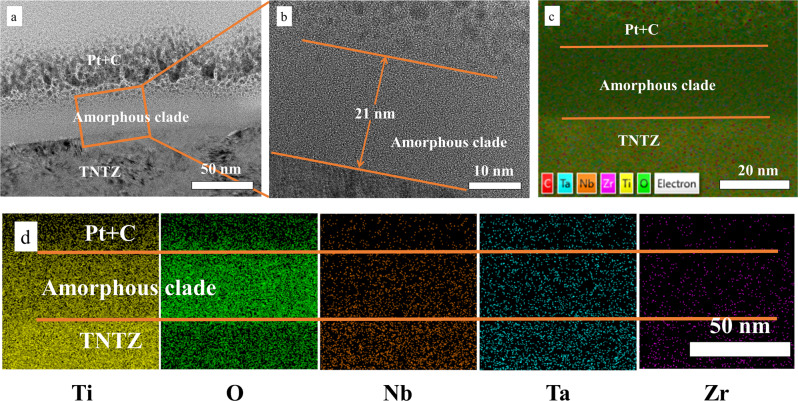
The cross section image of electropolished TNTZ. **a** FIB spectra, (**b**) High Resolution Transmission Electron Microscope (HRTEM) spectra, (**c**) EDS layer image, (**d**) Kα of Ti, O, Nb, Ta, Zr

The cross-section image of electropolished TNTZ (Fig. 3a, b) shows that the thickness of the amorphous clade was ~21 nm. It was possible that H^+^ in the acidic electrolyte transformed the β-phase of Ti-Nb alloy into an amorphous state [13, 14]. Figure 3c shows that all components of the alloy had diffused in the amorphous coating. EDS layer image (Fig. 3d) indicates that, in the amorphous coating, the content of O increased, while the content of Ti and Nb decreased greatly.

**Fig. 4:**
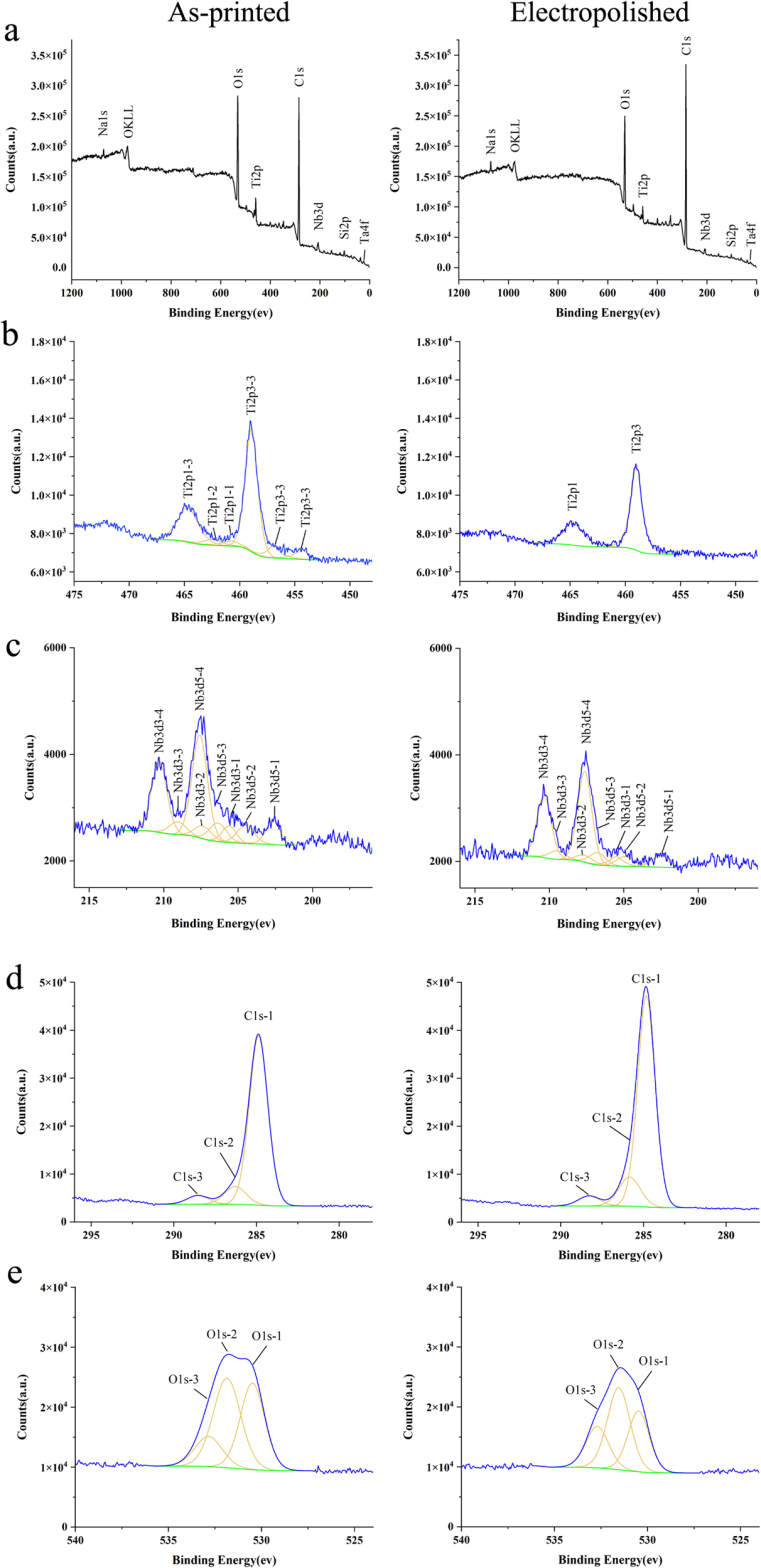
XPS analyses of (**a**) as-printed TNTZ and electropolished TNTZ. Fine analyses of (**b**) Ti element, (**c**) Nb element, (**d**) C element, (**e**) O element

Figure 4a, b shows the XPS survey spectra at binding energies (BEs) of 0–1350 eV for the TNTZ sample, which was sputtered using an Ar-ion beam for 0 s. The surface of TNTZ sample mainly consisted of TiO_2_ and Nb_2_O_5_, while the content of ZrO_2_ and Ta_2_O_5_ was much lower. The narrow spectrum (Fig. 4c, d) shows that the atomic ratio of Ti^4+^ and Nb^5+^ decreased greatly after electropolishing. The narrow spectrum (Fig. 4e, f) indicates that the residual C-C/C-H and some metal oxides on the surface of the material were transformed into C-O, C = O [15], which was key to the biocompatibility of biomedical Ti alloys. However, electropolishing could make the as-printed TNTZ samples showing differences in protein adsorption and cell behavior compared to the conventional TiO_2_ films [16].

### In vitro biocompatibility

**Fig. 5:**
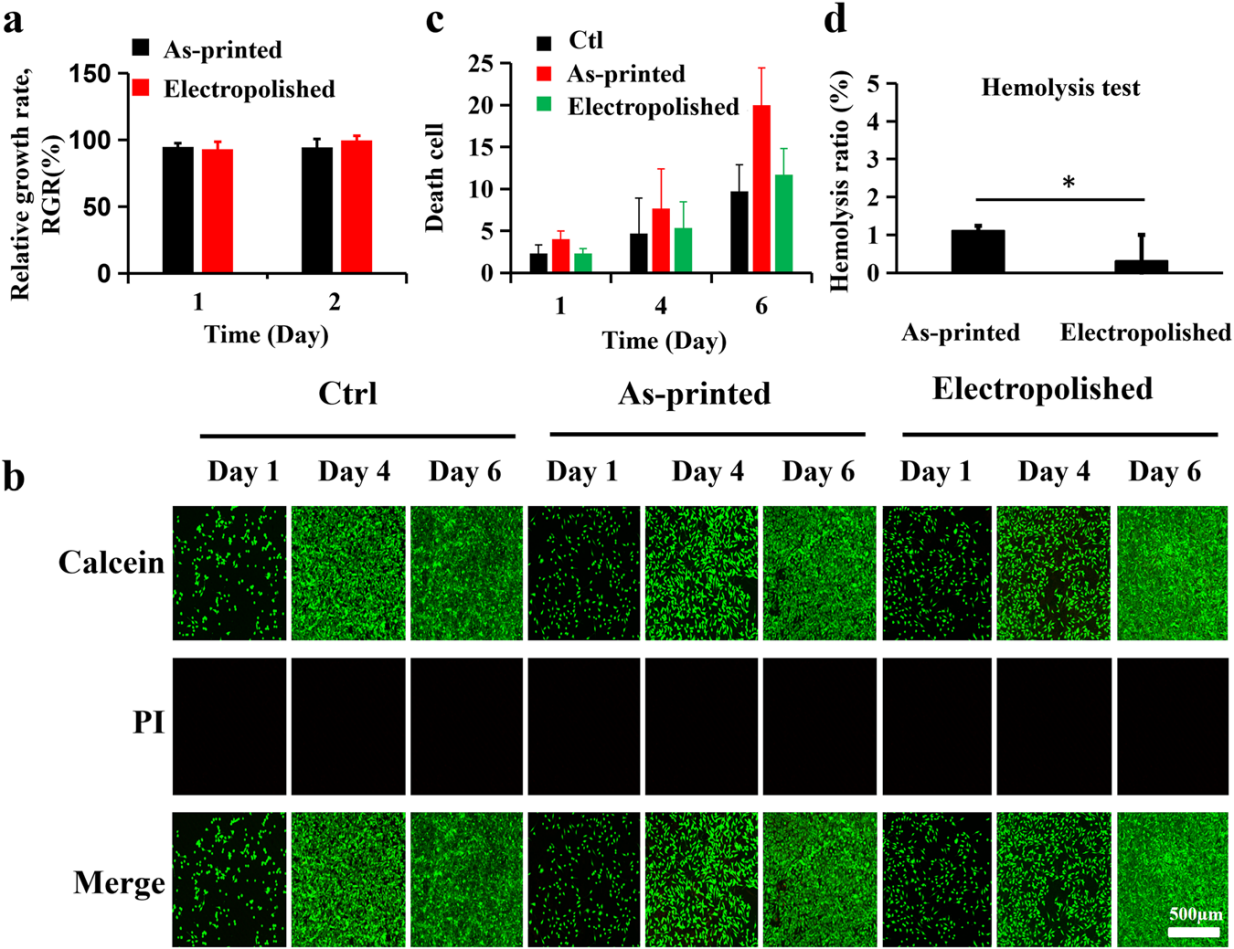
Effects of TNTZ on viability in L929 cells. **a** CCK-8 test of the TNTZ extracts solution, (**b**) the live and dead cells were detected by Calcein-AM/PI double staining, (**c**) dead cell statistics ×10 objective magnification, (**d**) hemolysis. Data were presented as Mean ± SD, *n* = 3

As shown in Fig. 5a, the cytotoxicity of TNTZ extracts solution was tested by CCK-8, the viability of L929 cells gradually increased from 24 to 48 h. Moreover, the cell viability of the electropolished group was better than the as-printed group. To assess the cell viability, L929 cells were stained with Calcein (AM, green, live cells) and propidium iodide (PI, red, dead cells). As shown in Fig. 5b, green fluorescence representing living cells markedly increased with the time. However, very little red fluorescence representing dead cells was observed in all groups. As shown in Fig. 5c, Image J analysis system was used to analyze the cells dyed red by PI in each photo, the results showed that all groups showed an increased number of cells dead over time. The electropolished TNTZ group showed the less number of dead cells than as-printed TNTZ, but higher than the control group of cell death and the as-printed TNTZ group stood in the highest position. These results indicated that electropolished TNTZ hardly inhibited the proliferation of L929 cells. The results of the hemolysis test were shown in Fig. 5d. The hemolysis ratio in as-printed TNTZ and electropolished TNTZ was (1.1 ± 0.14)% and (0.3 ± 0.7)%, respectively, lower than the standard hemolysis ratio (5%), hemolysis ratio of electropolished TNTZ exhibiting a great difference to the as-printed TNTZ, which indicates that these two kinds TNTZ conform to the given standard [17].

### In vivo biocompatibility and inflammatory response

**Fig. 6:**
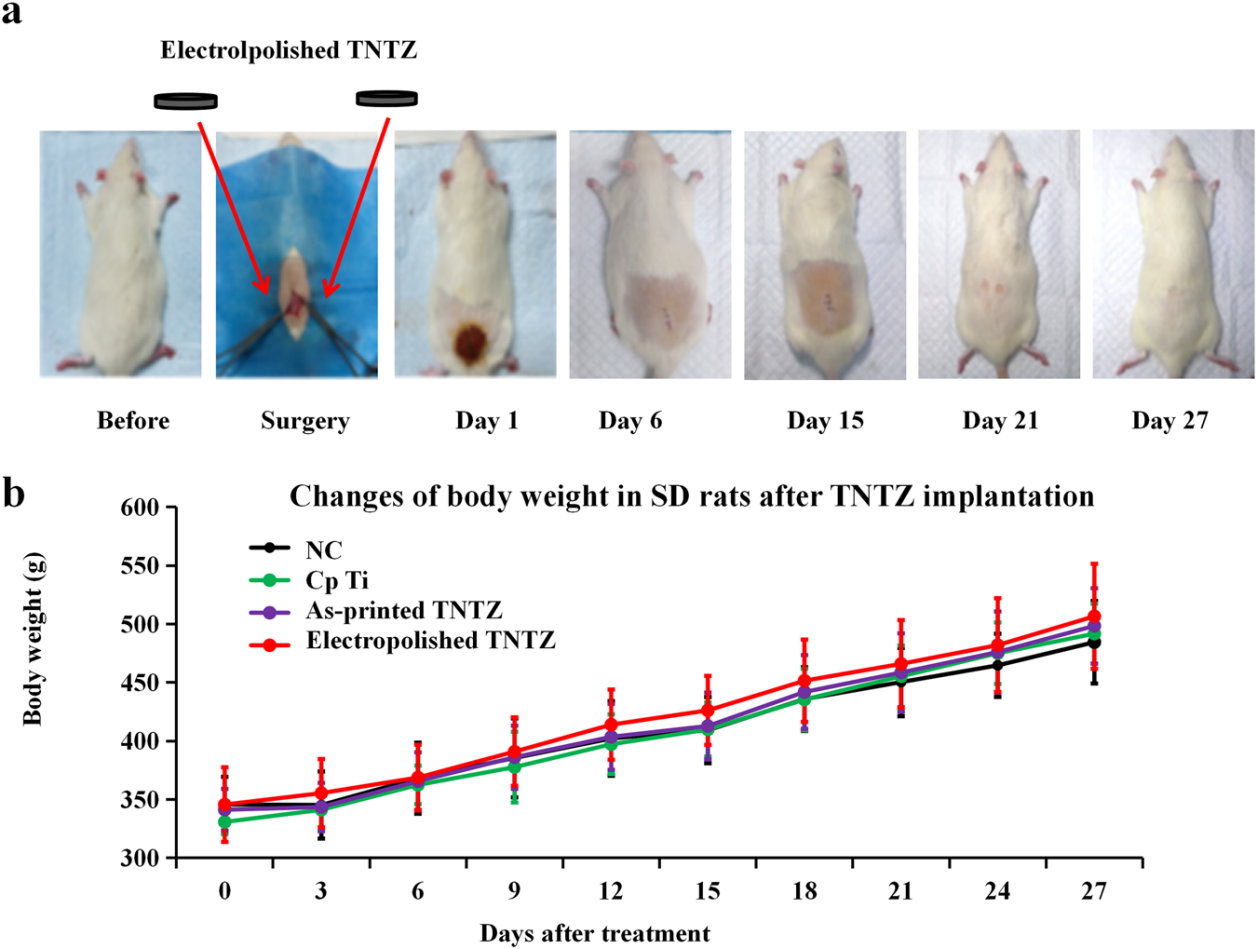
In vivo implantation of metal sheets as-printed and polished surface modification. **a** Postoperative wound changes of SD rats in group electropolished TNTZ. **b** Body weight changes of animals in each group 4 weeks after operation

To study the biocompatibility of different samples with soft tissue, subcutaneous implantations of titanium alloy sheets in a rat model was carried out, as schematically shown in Fig. 6a. All samples were both-side modified, meanwhile, Cp Ti was used as the positive control group. The control group underwent the same procedure, but did not implant any material. After implantation for 4 weeks, the incisions on the back were completely healed and the back hair had grown normally, and all the specimens implanted into the back did not move or fall, the other surgical pictures were shown in Supplementary Material. The 4-week body weight records showed that the body weight of each group increased steadily, indicating that the surgery and implant TNTZ had no obvious toxicity effect on the life function of SD rats Fig. 6b. Further study (Table 5) found that after 4 weeks of implantation, no elements were detected in the muscle tissue around the electropolished TNTZ. However, 0.187 μg/kg of Zr and 0.132 μg/kg of Ta was found in the muscle tissue around the as-printed TNTZ, indicating that the electropolished TNTZ had better stability and biological safety.

**Table 5:** Muscle ion precipitation around implants

Group	Sample	Ion precipitation (μg/kg)			
		Ti	Nb	Ta	Zr
1	Control	0	0	0	0
2	Cp Ti	0	0	0	0
3	As-printed TNTZ	0	0	0.132	0.187
4	Electropolished TNTZ	0	0	0	0

**Fig. 7:**
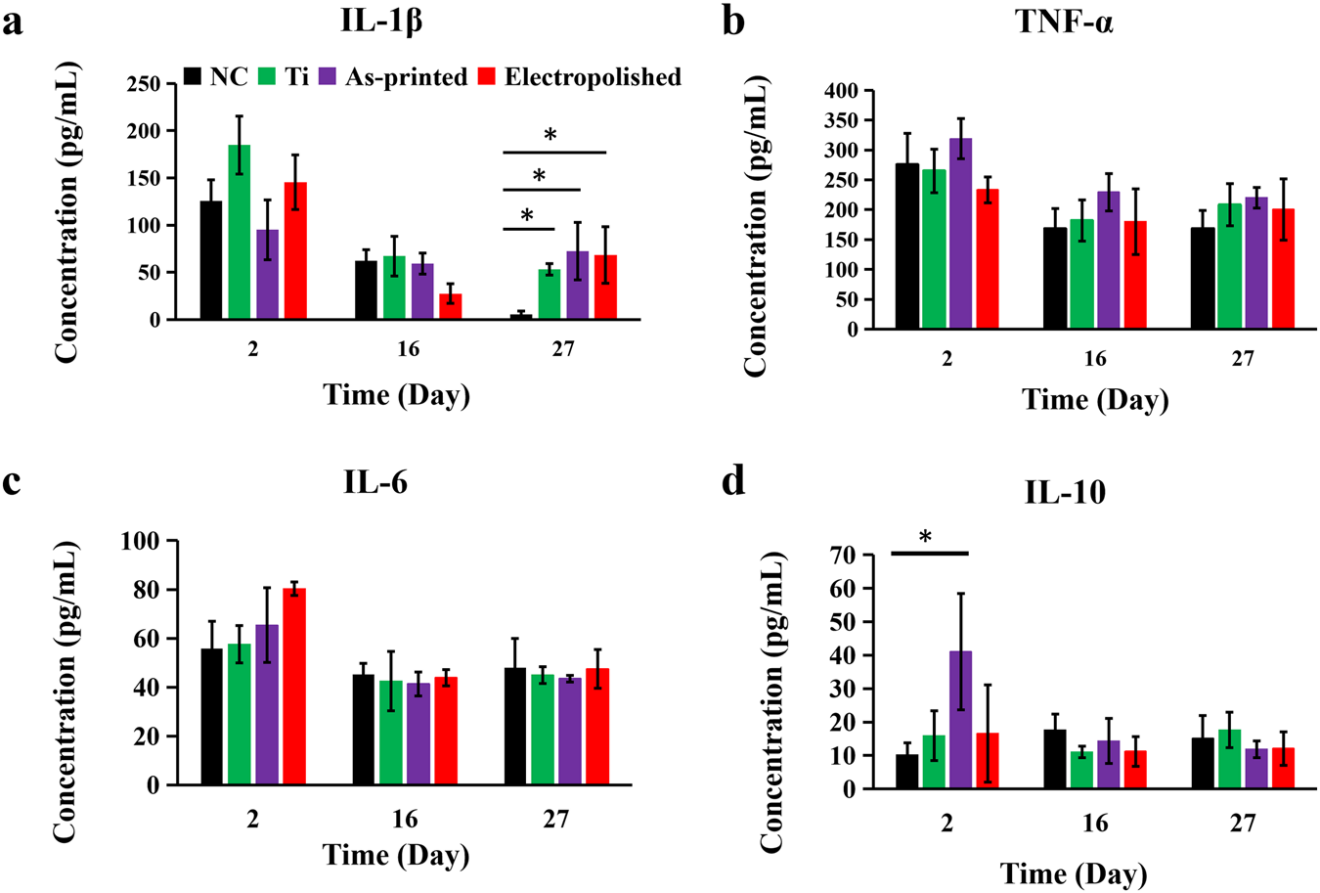
The levels of inflammatory factors in SD rats serum were quantified by ELISA. **a** IL-1β; (**b**) TNF-α; (**c**) IL-6; (**d**) IL-10

To analyze the inflammatory response induced by implants, changes in the levels of pro-inflammatory cytokines (IL-1β, TNF-α, IL-10), and anti-inflammatory cytokine (IL-6) in the serum of SD rats were detected. Serum was collected on days 2, 16, and 27 after implantation to analyze the immune response induced by the implants. The results showed fluctuations in the expression levels of pro-inflammatory and anti-inflammatory cytokines in each group. As shown in Fig. 7, on day 2 after surgery, compared with the NC group, the expression levels of IL-1β, IL-6, and IL-10 in the Cp Ti group increased, while the expression level of TNF-α decreased, but there was no significant difference. The expression levels of TNF-α, IL-6, and IL-10 increased in the as-printed TNTZ group, while IL-1β decreased, and there was a significant difference in the expression of IL-10. The expression levels of IL-1β, IL-6, and IL-10 increased in the electropolished TNTZ group, while TNF-α decreased, but there was no significant difference. Analysis of data on days 16 and 27 showed that the expression levels of each cytokine tended to balance, but the expression of IL-1β in the Cp Ti, as-printed TNTZ, and electropolished TNTZ groups was much higher than that in the NC group (showed significant differences). After the implantation, the presence of the implant may cause local inflammatory and immune reactions. At this time, the increased content of IL-10 in the SD rat body may indicate that the immune system was regulating and suppressing the occurrence of inflammatory reactions to reduce tissue damage and pain symptoms. In addition, the increase in IL-10 may also help promote the repair and regeneration of tissues around the bone nail. IL-1β was produced by immune cells such as monocytes, macrophages, and dendritic cells. It can promote inflammatory reactions, migration and activation of immune cells. It also can stimulate reactions in the brain, endocrine, and nervous systems. After a certain period of retention in SD rats, the body recognizes the implant as a foreign object and secretes IL-1β to promote an inflammatory response to clear foreign objects from the body.

**Fig. 8:**
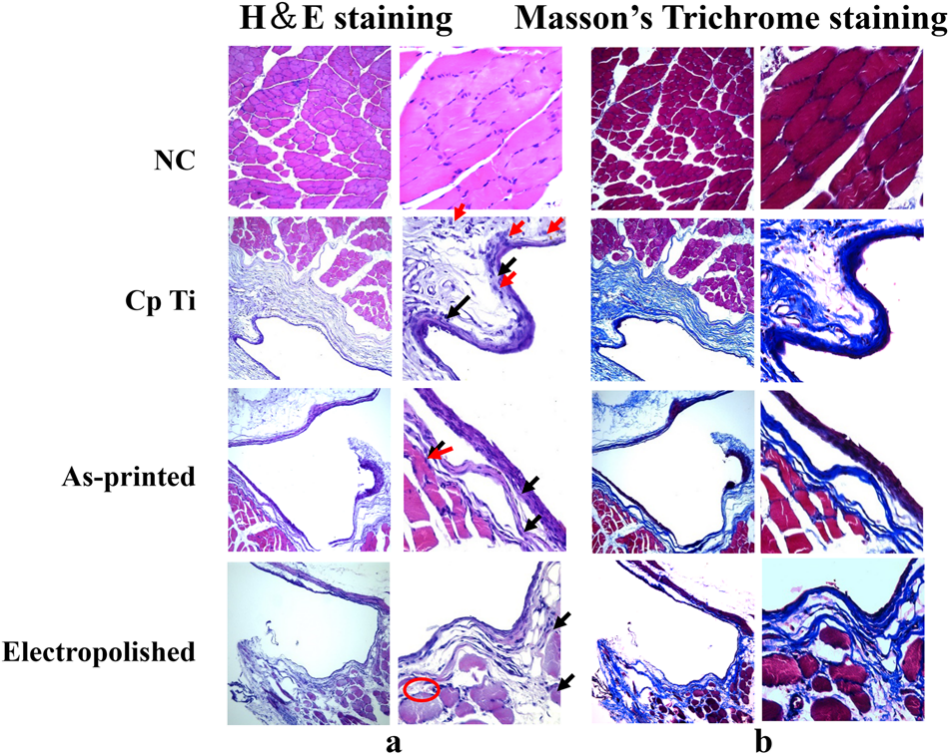
Histological analysis of tissue after 4 weeks implantation in SD rats, (**a**) H&E staining, (**b**) Masson’s Trichrome staining

The histological images of fibrous capsulation around Cp Ti and TNTZ implants were observed with results as shown in Fig. 8. The HE staining images of the tissue at 4 weeks were presented in Fig. 8a. The control group, without implants, recovered well and formed intact muscle tissue. All the Ti and TNTZ groups could find fibrous capsules, the formation of capsule thickness was the thickest in Ti, followed by as-printed TNTZ and the thinnest in electropolished TNTZ. Inflammatory cells were also observed with macrophages marked in red and lymphocytes marked in black. It appeared that the inflammatory cells density were also the highest in Cp Ti, followed by as-printed TNTZ and electropolished TNTZ, neovascularization was also observed in electropolished TNTZ, marked red circle. Masson’s staining images present a similar result as shown as Fig. 8b, more new tissue could be seen in the electropolished TNTZ, and electropolished TNTZ showed less inflammatory cell infiltration, no matter compared to the unpolished or pure titanium group. Compared to Cp Ti, electropolished TNTZ could also decrease the thickness of fibrous capsulation, and promote the formation of new blood vessels, while there were still many inflammatory cells.

## Discussion

### Inflammation analysis

From the above results, it could be seen that electropolished TNTZ present excellent biosafety and could promote tissue regeneration. The thickness of the fibrous capsule was an indicator to evaluate the histocompatibility of an implant [18]. It was clear that when any biomedical device was implanted into mammals, it would cause a typical inflammatory reaction [19], consisting of diverse cell signaling and complex inflammatory reaction, followed by migration of fibroblasts to the surface of the implant and the subsequent differentiation of smooth muscle actin-expressing myofibroblasts. These cells then deposited a collagen capsule that surrounds and effectively walls off the device from the surrounding soft tissues [20]. At some point, myofibroblasts within the capsule could initiate contraction with resulting distortion, migration, and seeming firmness of an originally soft device [21].

The ELISA results indicated that different materials have different effects on SD rats. As shown in Fig. 8a, from HE staining, it can be seen that macrophages and lymphocytes were distributed around the implants after 4 weeks, with the lowest number of inflammatory cells in the electropolished group. Implantation of medical devices can cause an immune response, leading to the aggregation of immune cells such as macrophages and lymphocytes. This aggregation was part of the body’s immune defense response to the implant, and they attempted to clear the foreign objects around the implant to maintain body homeostasis, which was a beneficial immune response to the body. Over time, the immune response gradually weakened, but the aggregation of immune cells around the implant may still existed [22]. Due to the long-term stimulation of the implant, the aggregation of immune cells secreted cytokines, stimulating fibroblast proliferation and collagen production, promoting the formation of fibrous capsules. Furthermore, the secretion of fibronectin, collagen, and other matrix components provide structural support for the formation of fibrous capsules. The new tissue around orthopedic implants was a double-edged sword in clinical practice [11]. Appropriate growth factors and cytokines could promote the interaction between the implant and surrounding tissues, which was beneficial for tissue repair and regeneration [23]. As shown in Fig. 8b, Masson’s Trichrome staining showed that the fibrous capsules of the pure titanium and as-preinted groups were relatively thick, but the boundary between the fibrous capsule and the new muscle tissue was clear, while the electropolished group had only a thin fibrous capsule and tight muscle tissue, indicating that the implant of the electropolished group could be firmly combined with the surrounding tissue. When the implant enters the body, the body will produce an immune response, which forms a certain degree of inflammatory reaction through various pathways. The inflammatory reaction will cause local vascular dilation and increased vascular permeability, thereby promoting the migration and proliferation of local tissue cells. At the same time, the body will also release various growth factors, such as fibroblast growth factor (FGF), vascular endothelial growth factor (VEGF), etc., which could stimulate angiogenesis and cell proliferation, thereby forming new tissue. The formation of new tissue could improve the connection between the implant and the surrounding tissue to a certain extent, improve the stability and function of the implant. Combining HE staining and Masson’s Trichrome staining, the new blood vessels in the electropolished group were the most evident.

### Proteomics analysis

Proteomics was conducted to examine interactions between proteins of the L929 cell and TNTZ discs. In total, 4962 proteins were identified in the proteomic results. Specifically, 57 differentially expressed proteins (significant changing differences: 30, Consistent presence/ absence expression differences: 27) were identified in the L929 cells for the electropolished TNTZ.

#### Differential expression analysis

**Fig. 9:**
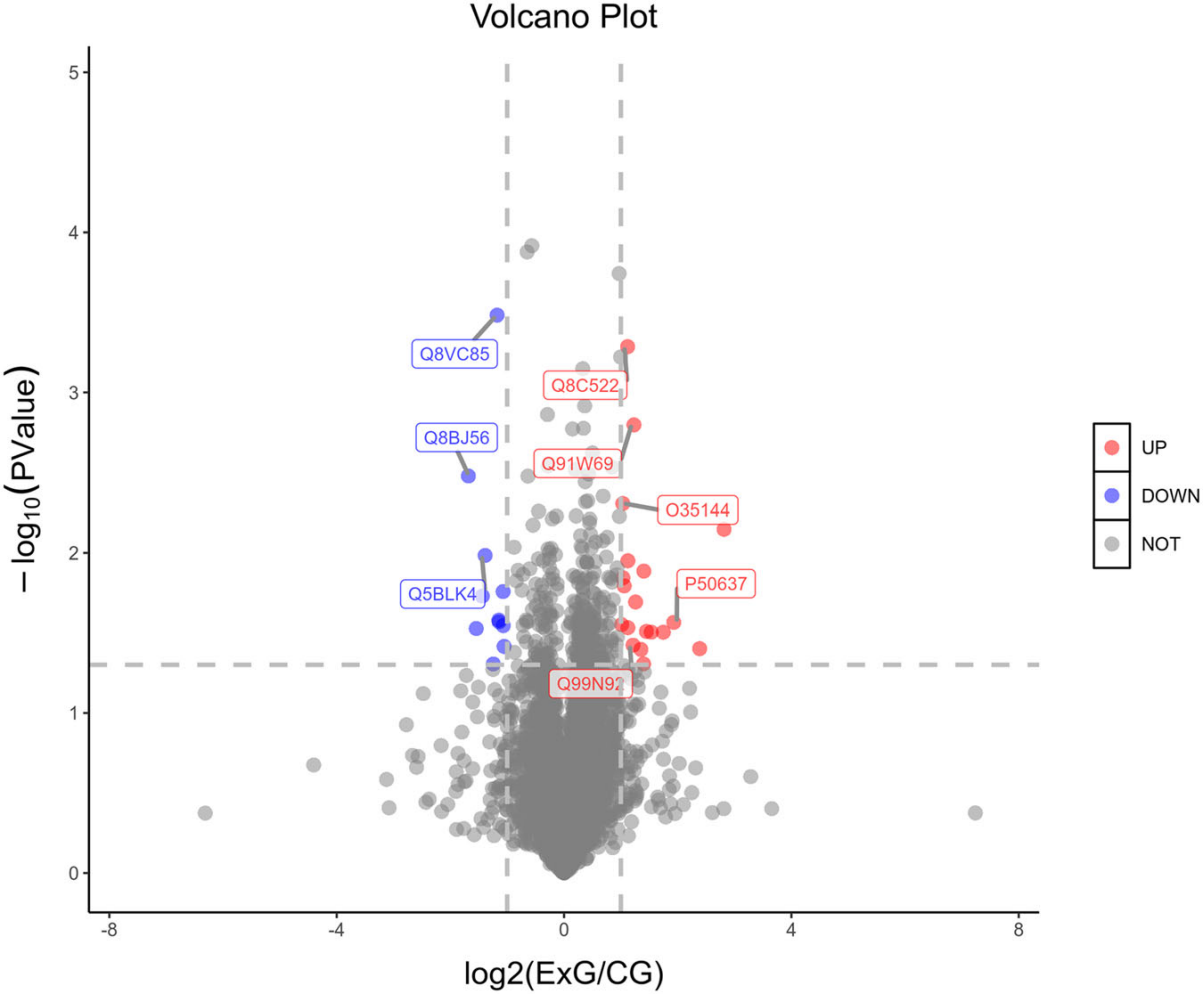
Volcano diagram of the differential expressed proteins

After analysis, there were 19 up-regulated proteins and 11 down-regulated proteins. Figure 9 suggests that the fold change of the up-regulated proteins were more significant (up-regulated: 2–3 times, down-regulated: 2–2.5 times). It was noted that among all the differentially expressed proteins, Q8C522 was the most up-regulated in the electropolished TNTZ when compared to the as-printed TNTZ. The Q8C522 aslo called endonuclease domain-containing 1 protein, which may act as a DNAse and a RNAse [24]. Similarly, P50637 and 17 other differentially expressed proteins were also up-regulated. Figure 9 also indicates that 11 differentially expressed proteins were down-regulated, including the Q8VC85. Q8VC85 aslo called U6 snRNA-associated Sm-like protein LSm1, which played a role in the degradation of histone mRNAs, the only eukaryotic mRNAs that were not polyadenylated (by similarity), and probably also the part of an LSm subunits-containing complex involved in the general process of mRNA degradation (by similarity) [25].

In addition, “Consistent presence/ absence expression profile” difference comparison was used (at least two samples in one group were not null, and all in the other group were null), and 23 up-regulated and 4 down-regulated differential proteins were additionally detected (Table 6). Due to the lack of data from the third test, the “whether or not”comparison results were not involved in cluster analysis and volcano map drawing, only as a reference for subsequent protein function analysis.

**Table 6:** Statistics of quantitative results of differentially expressed proteins

Comparisons	Significantly changing in abundance		Consistent presence/absence expression profile		Total
Electropolished vs As-printed	Up-regulated	Down-regulated	Up-regulated	Down-regulated	
	19	11	23	4	57

#### GO function analysis

**Fig. 10:**
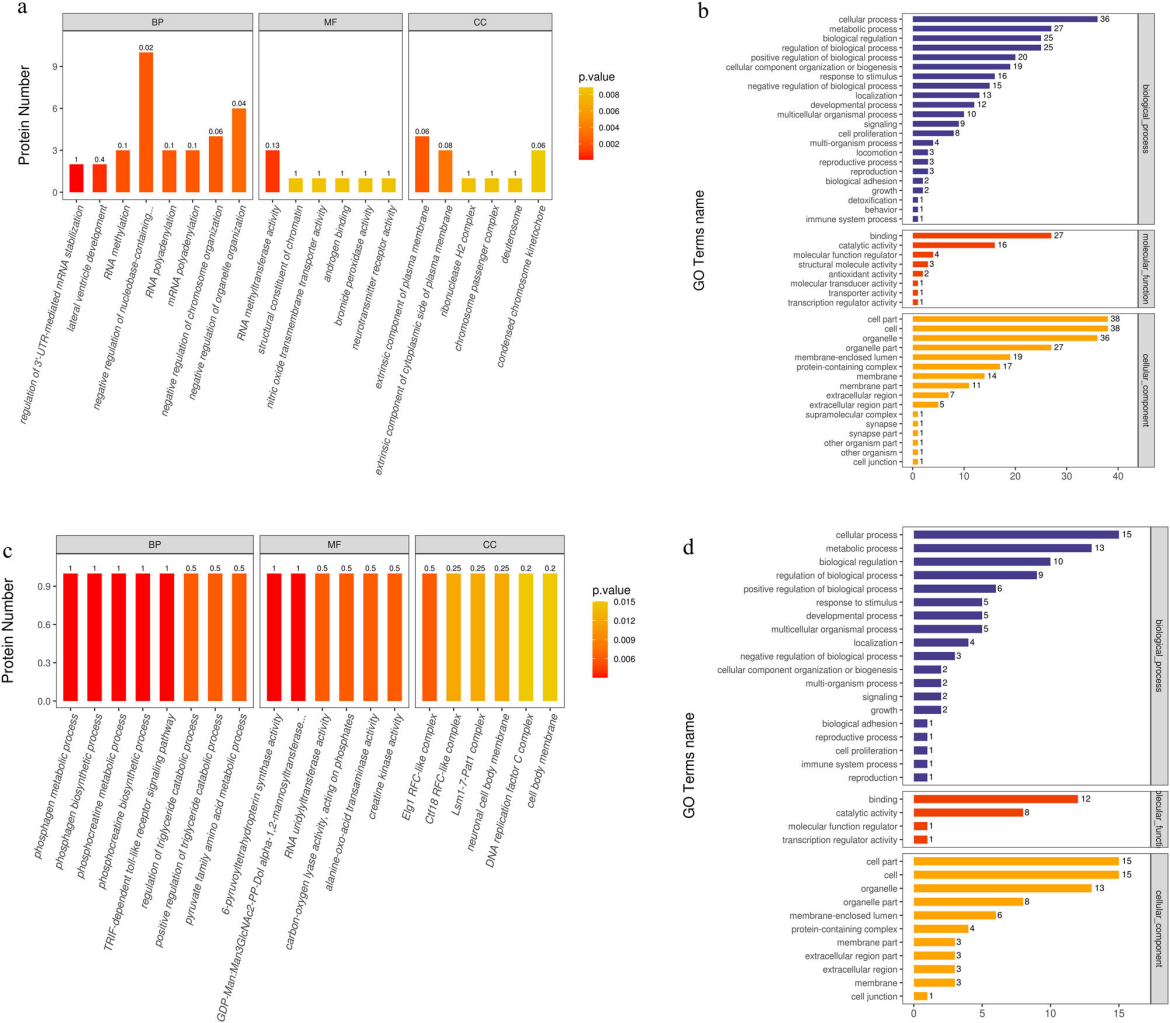
GO enrichment analysis of electropolished TNTZ: (**a**) the up-regulated group level 1, (**b**) the up-regulated group level 2, and (**c**) the down-regulated group level 1, (**d**) the down-regulated group level 2. In figure (**a**) and (**c**), the lower the *P* value, the more obvious the expected phenomenon

The differentially expressed proteins were further divided into two groups, namely the up-regulated and down-regulated groups. To fully understand the differences in protein function in the organism, positioning and participation in biological pathways, Blast2Go [https://www.blast2go.com/] software was adopted to make GO functional annotation, and the number of differentially expressed proteins was calculated in the secondary functional annotation level (Fig. 10).

- Figure 10a shows the signific antly up-regulated groups (*p* < 0.05) in electropolished TNTZ, compared with as-printed TNTZ. Based on GO function analysis, they are linked to many GO terms. Several biological processes (BPs) were detected in the group, including regulation of 3ʹ-UTR-mediated mRNA stabilization, lateral ventricle development, RNA methylation, negative regulation of nucleobase-containing compound metabolic process, RNA polyadenylation, mRNA polyadenylation, negative regulation of chromosome organization, and negative regulation of organelle organization. Most of them had influenced the metabolism of methyl-transferase and base compound (Fig. 10b). A variety of molecular functions (MFs) were also detected, including RNA methyltransferase activity, structural constituent of chromatin, nitric oxide transmembrane transporter activity, androgen binding, bromide peroxidase activity, and neurotransmitter receptor activity. Among them, RNA methyl-transferase activity appears as the most significantly enriched MF, thereby suggesting that the electropolished TNTZ better promotes RNA methyl-transferase. 6 cellular components (CCs), such as extrinsic component of the membrane and extrinsic component of cytoplasmic side of plasma membrane, were detected. This indicated that the electropolished TNTZ implant might have a great effect on the extrinsic component of L929 cells.
- Figure 10c shows the significantly down-regulated groups (*p* < 0.05) in the electropolished TNTZ, and compared with the as-printed TNTZ. The BPs corresponded to phosphagen metabolic process, phosphagen biosynthetic process, phosphocreatine metabolic process, phosphocreatine biosynthetic process, TRIF-dependent toll-like receptor signaling pathway, regulation of triglyceride catabolic process, positive regulation of triglyceride catabolic process, and pyruvate family amino acid metabolic process. Some of them influenced the phosphate synthesis and metabolism, and then affected cell proliferation and adhesion (Fig. 10d). The MFs detected in the group include 6-pyruvoyltetrahydropterin synthase activity, GDP-Man:Man3GlcNAc2-PP-Dol alpha-1,2-mannosyltransferase activity, RNA uridylyltransferase activity, carbon-oxygen lyase activity, acting on phosphates, alanine-oxo-acid transaminase activity and creatine kinase activity. 6 CCs were detected, including Elg1 RFC-like complex, Ctf18 RFC-like complex, Lsm1-7-Pat1 complex, neuronal cell body membrane, DNA replication factor C complex, and cell body membrane.

#### KEGG pathway analysis

**Fig. 11:**
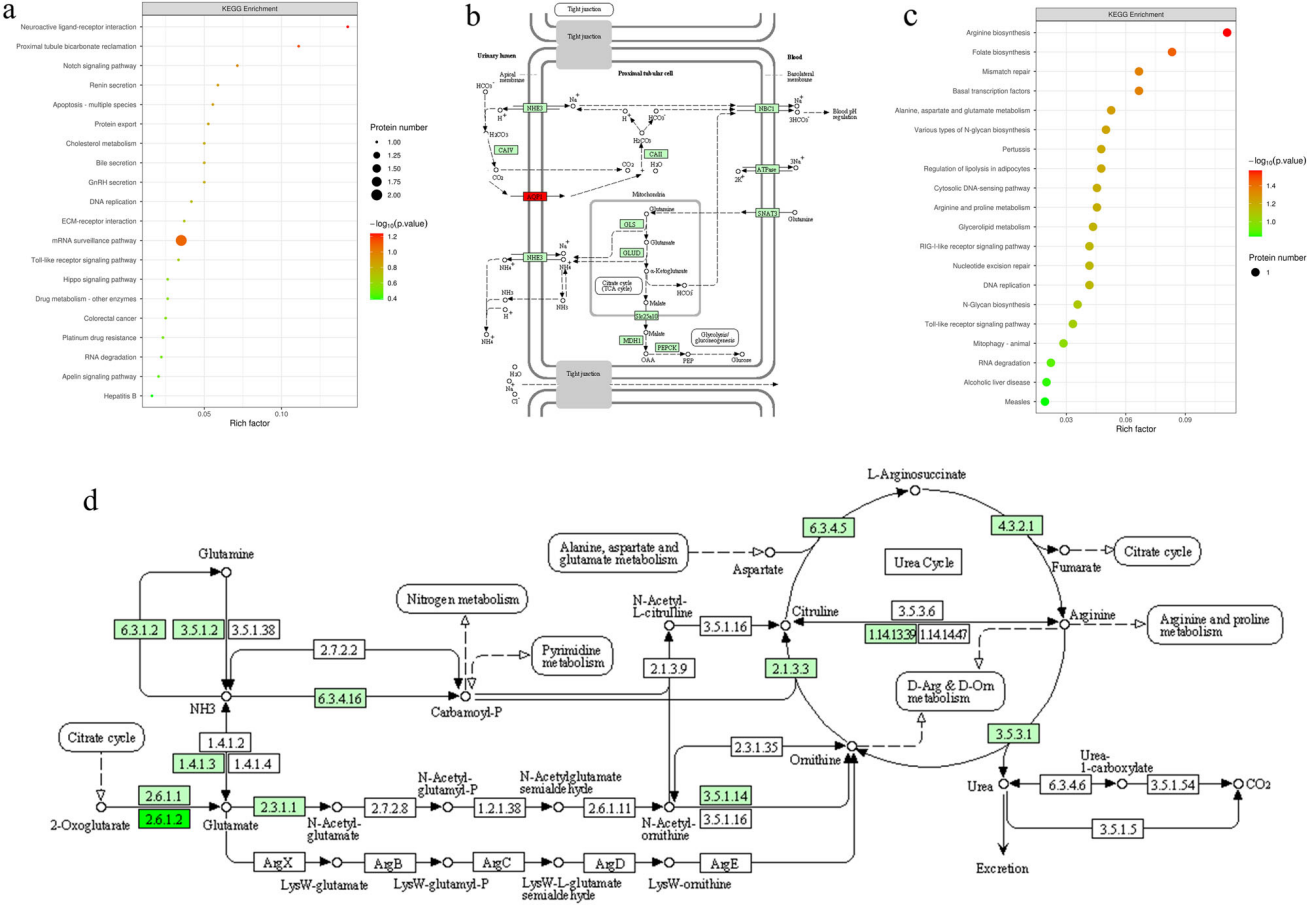
The KEGG enrichment analysis of electropolished TNTZ: (**a**) the up-regulated group, (**b**) schematic diagram of Proximal tubule bicarbonate reclamation signal pathway obtained from the KEGG database with some modifications (red color represents upregulation proteins), and (**c**) the down-regulated group, (**d**) schematic diagram of Arginine biosynthesis signal pathway obtained from the KEGG database with some modifications. In figure (**a**) and (**c**), the lower the *P* value, the more obvious was the expected phenomenon

To analyze more systematically and comprehensively, Fisher’s exact test was used to analyze KEGG pathway enrichment of up-regulated and down-regulated groups, respectively (Fig. 11).

- Concerning the up-regulated group, Fig. 11a illustrates that important pathways, such as the Neuroactive ligand-receptor interaction, Proximal tubule bicarbonate reclamation, and mRNA surveillance pathway were changed significantly when the electropolished TNTZ was compared to the as-printed TNTZ. Figure 11b shows that the electropolished TNTZ induced up-regulation of Aquaporin (AQP1), which might played an important role in the reabsorption of water [26]. Therefore, electropolished TNTZ could increase the activity of cells.
- For the down-regulated group, Fig. 11c detects important pathways, including Arginine biosynthesis, and Folate biosynthesis. L-arginine was an essential amino acid for bone and skeletal muscle growth. Studies have shown that, arginine promotes tissue growth by promoting growth hormone secretion in the pituitary gland [27]. In other words, when arginine synthesis is reduced, tissue growth would be delayed. Figure 11d shows that electropolished TNTZ inhibited arginine synthesis-related pathways and inhibits tissue hyperplasia.

Some studies have shown that metal materials can affect adherent cells by adsorbing proteins from the external environment [28]. It shows that electropolished TNTZ could adsorb some proteins and hinder ion precipitation

## Conclusions

- 30% oxalic acid electropolishing was effective in solving balling defects, and a ~21 nm amorphous clad layer formed on the surface of the material after polishing.
- According to the in vitro biocompatibility evaluation, the electropolished TNTZ showed similar cell cytotoxicity and better blood biocompatibility than the as-printed TNTZ.
- The animal experiments showed that the amorphous clad layer could make a battier to prevent Ta and Zr ions from penetrating the muscle tissue, and there could form a good tissue regeneration at the implantation site during 4 weeks, indicating that the electropolished TNTZ has good potential as biomedical implant.
- The results of proteomics results suggest that the electropolished TNTZ can significantly effect on RNA transcription of L929 cells, changing the pathways related to cell synthesis, metabolism and catalysis, making the cells show higher antioxidant capacity (longer life) but less proliferation, than those attached to the as-printed TNTZ.

## Supplementary information

Supplementary Material
