# Supplementary figures and images for: Electropolishing influence on biocompatibility of additively manufactured Ti-Nb-Ta-Zr: in vivo and in vitro
Source: J Mater Sci Mater Med. 2023 May 14;34(5):25. doi: 10.1007/s10856-023-06728-0 (PMC10183419; doi:10.1007/s10856-023-06728-0)

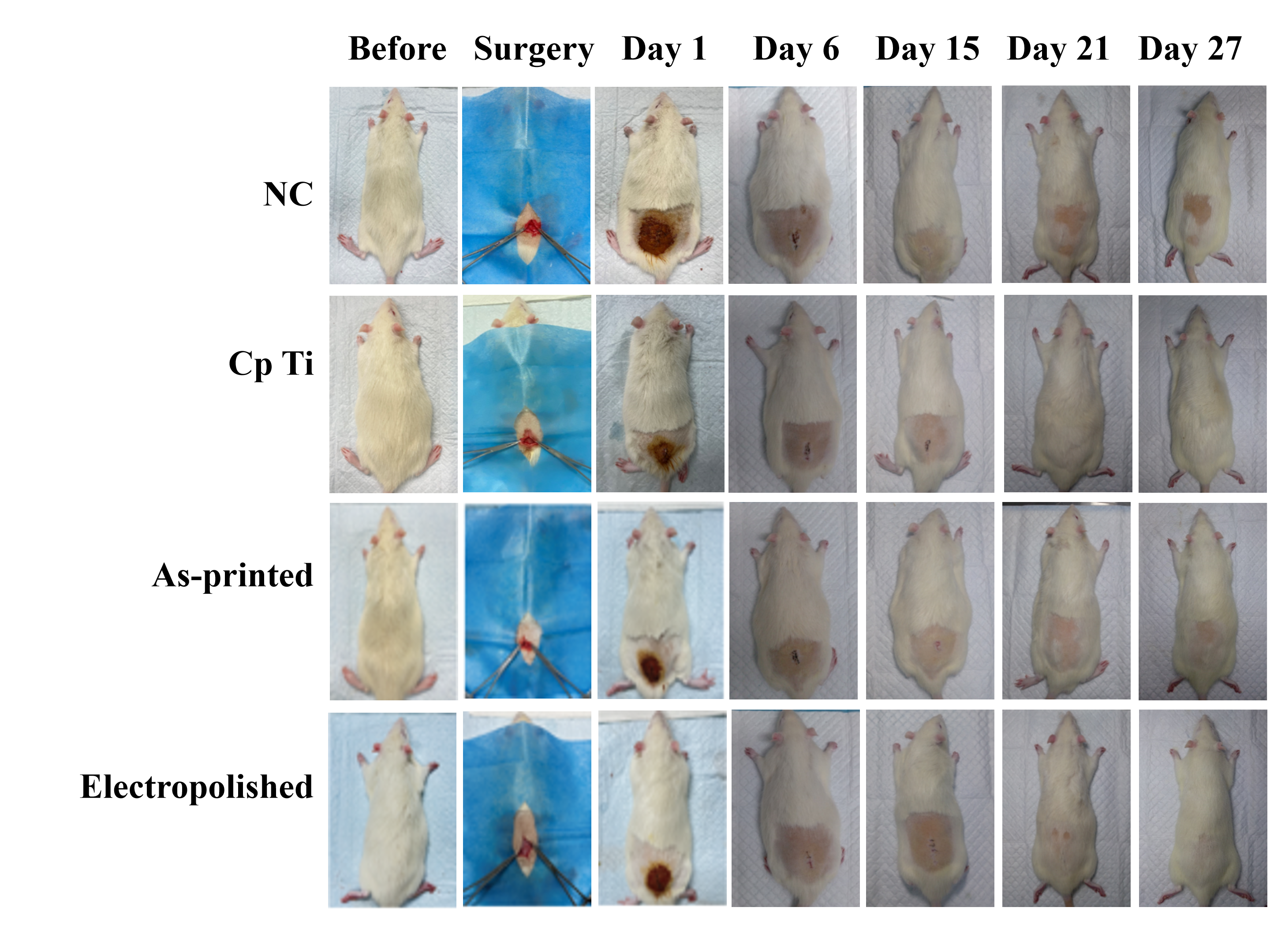

Supplement: Supplementary file 1 — Supplementary Material [file 10856_2023_6728_MOESM1_ESM.png]
